# Supplementary material for: MetaRibo-Seq measures translation in microbiomes
Source: Nat Commun. 2020 Jun 29;11:3268. doi: 10.1038/s41467-020-17081-z (PMC7324362; doi:10.1038/s41467-020-17081-z)
Supplement: Supplementary file 10 — Supplementary Data 7 [file 41467_2020_17081_MOESM10_ESM.zip › File2/Confidence_High_Taxonomy/58014_out.krona.html]

Javascript must be enabled to view this page.

members
magnitude
magnitudeUnassigned
count
unassigned
taxon
rank

58014\_out

49

49
2
superkingdom

1239
49
phylum

186801
49
class

186802
49
order

family
49
186803


SRS011405\_contig\_number\_9112SRS012273\_contig\_number\_contig-100\_845.256924SRS014855\_contig\_number\_contig-100\_241.109029SRS015663\_contig\_number\_contig-100\_9.212984SRS015694\_contig\_number\_19248SRS015854\_contig\_number\_17726SRS016335\_contig\_number\_47941SRS016989\_contig\_number\_contig-100\_541.101885SRS017433\_contig\_number\_450SRS019496\_contig\_number\_3262SRS020233\_contig\_number\_18491SRS024009\_contig\_number\_11735SRS043001\_contig\_number\_11645SRS047014\_contig\_number\_32687SRS047044\_contig\_number\_18741SRS048870\_contig\_number\_contig-100\_8596.52361SRS050026\_contig\_number\_contig-100\_9739.46078SRS051031\_contig\_number\_contig-100\_3.198959SRS052027\_contig\_number\_18366SRS056259\_contig\_number\_30736SRS062701\_contig\_number\_7373SRS065504\_contig\_number\_18889SRS075716\_contig\_number\_4704SRS076756\_contig\_number\_contig-100\_732.94562SRS076804\_contig\_number\_15606SRS077502\_contig\_number\_contig-100\_2102.48331SRS077552\_contig\_number\_contig-100\_7814.49424SRS098514\_contig\_number\_contig-100\_1077.1077SRS098644\_contig\_number\_47113SRS1041133\_contig\_number\_24424SRS1041136\_contig\_number\_7352SRS1041142\_contig\_number\_8560SRS104311\_contig\_number\_31264SRS104400\_contig\_number\_60824SRS104693\_contig\_number\_6755SRS1055038\_contig\_number\_4967SRS1055083\_contig\_number\_contig-100\_15686.15687SRS142542\_contig\_number\_16849SRS142781\_contig\_number\_9636SRS143342\_contig\_number\_17170SRS147346\_contig\_number\_42124SRS148159\_contig\_number\_40932SRS149181\_contig\_number\_31986SRS893270\_contig\_number\_20247SRS893295\_contig\_number\_10668SRS893300\_contig\_number\_11377SRS893342\_contig\_number\_4601SRS893366\_contig\_number\_24693
48
1898203
species

genus
841
1

species
1
2049040

SRS062654\_contig\_number\_26553
